# A Comparative Study of Temporomandibular Joints in Adults with Definite Sleep Bruxism on Magnetic Resonance Imaging and Cone-Beam Computer Tomography Images

**DOI:** 10.3390/jcm12072570

**Published:** 2023-03-29

**Authors:** Juan Zhang, Wenjuan Yu, Jianghong Wang, Sijia Wang, Yifan Li, Huimin Jing, Zekui Li, Xin Li, Meng Liang, Yonglan Wang

**Affiliations:** 1 Department of Prosthodontics, Tianjin Medical University School and Hospital of Stomatology, Tianjin 300070, China; 2 Department of Stomatology, The Seventh Affiliated Hospital, Sun Yat-Sen University, Shenzhen 518100, China; 3 School of Medical Imaging and Tianjin Key Laboratory of Functional Imaging, Tianjin Medical University, Tianjin 300070, China; 4 Department of Radiology, Tianjin Medical University School and Hospital of Stomatology, Tianjin 300070, China; 5 Department of Periodontics, Tianjin Medical University School and Hospital of Stomatology, Tianjin 300070, China

**Keywords:** temporomandibular joint disorders (TMD), sleep bruxism, magnetic resonance imaging (MRI), cone-beam computer tomography (CBCT)

## Abstract

Objective: The aim was to study the imaging characteristics of the temporomandibular joint (TMJ) of definite sleep bruxers through magnetic resonance imaging (MRI) and cone-beam computer tomography (CBCT). Methods: Nineteen definite sleep bruxers diagnosed by polysomnography and twenty asymptomatic non-bruxers matched by age, gender, and education level participated in this study. After obtaining MRI and CBCT images of all TMJs of the subjects, evaluation and measurement were conducted, respectively. The analyzed parameters included disc position, disc configuration, joint effusion (JE), joint space or condyle position, and condylar bony changes. Results: Of the 38 joints in the study group, disc deformity and disc displacement of TMJs were both 57.9% when the mouth was closed, and 76.3% showed condylar bony changes, while when the mouth was open, 82% of all TMJs showed physiological biconcave discs. Comparison of joint space revealed that the anterior space was larger in the study group. There was no significant difference between the mild and the moderate to severe sleep bruxism subgroups in the changes of TMJ. Conclusion: The results demonstrated that a higher prevalence of disc deformity, disc displacement, JE, and condylar bony changes occurred in temporomandibular joints of sleep bruxers. These changes were not related to the severity of sleep bruxism.

## 1. Introduction

Sleep bruxism (SB) is a masticatory muscle activity during sleep that is characterized as rhythmic (phasic) or non-rhythmic (tonic) and is not a movement disorder or a sleep disorder in otherwise healthy individuals [1]. It is also characterized by clenching and grinding of the teeth during sleep [2]. Clenching is the forceful closure of the maxillary and mandibular dentition in a static relationship of the mandible to the maxilla in either maximum intercuspation or an eccentric position, and grinding is the forceful closure of the maxillary and mandibular dentition under the dynamic relationship when the mandible moves freely at different positions [3]. It differs from normal mastication in the constancy, the excess, and the excessive time each day of the force. It should be emphasized that clenching and grinding are not separate conditions but interact additively [4]. A systematic review has reported that the prevalence of SB was up to 12.8% in the adult population [5]. The gold standard for SB diagnosis is polysomnography (PSG), which is limited by the high costs and the small quantity of adequately equipped sleep laboratories. A study reported that during an SB episode, the masticatory muscle contraction activity greatly increases, and a considerable force will be absorbed by the structures such as teeth, articular disc, and condyle [6]. Once the activity exceeds the individual’s physiologic tolerance, the stomatognathic system will be altered and dysfunction may occur.

Temporomandibular disorder (TMD) is a group of complex disorders, involving the masticatory muscles, the temporomandibular joint (TMJ), and associated structures. The prevalence of TMD is high and a study pointed out that TMD was moderately prevalent (35%) [7]. Most researchers, including dentists and TMD experts, maintain that there is a connection between SB and TMD [8,9]. In the finite element analysis of TMJ, the shear stress generated during the clenching and grinding activity can induce damage in the articular disc, which in turn can lead to TMD [10]. However, a review found that there was no correlation between SB and TMD, although only for signs and symptoms of masticatory muscles [11]. Due to the complexity of etiology and diagnosis, there has always been controversy about the relationship between SB and TMD in the field of stomatology.

At present, there are few studies on TMJ radiology changes in sleep bruxers. Güler et al. [12] demonstrated that a high prevalence of condylar bony changes occurred in reducing joints in patients with bruxing behavior. It is worth emphasizing that magnetic resonance imaging (MRI) is not the best imaging method for evaluating the minor bone. Recently, Padmaja et al. [13] assessed the mandibular surface area changes on panoramic radiographic images and found that the surface area of condyle in bruxers had significantly changed compared with non-bruxers. It should also be pointed out that a panoramic radiographic image is not the best imaging method to evaluate the changes of condylar surfaces areas. Notably, these previous studies diagnosed SB only by self-report and/or clinical examination, and PSG was not used, resulting in low or moderate levels of evidence. So far, there is still a lack of relevant knowledge about the imaging characteristics of TMJ soft and hard tissues in definite sleep bruxers. Due to the high prevalence of SB and TMD, the study of TMJ imaging characteristics in SB patients is imperative.

Based on the aforementioned statements, this study enrolled SB patients diagnosed with PSG, and adopted the 3.0T MRI and cone-beam computer tomography (CBCT) imaging methods to comprehensively research the imaging characteristics of TMJ soft and hard tissues of definite SB patients by comparing them with asymptomatic non-bruxers, including the disc configuration, disc position, joint effusion (JE), joint space, and condylar bony changes, so as to provide a theoretical basis for the clinical treatment of TMD-SB patients. The study hypothesized that there are some differences in the imaging characteristics of TMJ soft and hard tissues of definite SB patients.

## 2. Materials and Methods

This study was approved by the Ethics Committee of the Stomatology Hospital of Tianjin Medical University. All the subjects voluntarily participated in and agreed to the whole examination procedures, including the questionnaires, clinical examination of TMJs, MRI, and CBCT scans of bilateral TMJs, and then signed an informed consent form; among them, only the patients with sleep bruxism underwent PSG. Moreover, the subjects could request to stop the relevant examinations at any time.

### 2.1. Sample Selection

We recruited volunteers from patients in the Stomatology Hospital of Tianjin Medical University and/or students from the School of Stomatology, of Tianjin Medical University, from October 2018 to June 2019, and finally included 39 subjects (8 males, 31 females, mean age 25.51 years, range 21–32 years). Among the subjects, 19 (4 males, 15 females) were diagnosed as SB by sleep specialists of Tianjin Medical University General Hospital through PSG, forming the study group. The other 20 subjects (4 males, 16 females) without bruxing behavior and without abnormalities of the TMJ according to the Research Diagnostic Criteria for Temporomandibular Disorders (RDC/TMD) [14] examination procedures formed the control group.

The exclusion criteria for both groups were as follows: (1) age out of the range of 20–35 years old, (2) history of treatment for TMD, (3) presence of asymmetric chewing, (4) current presence of orthodontic treatment, (5) presence of Angle’s Class II and Class III malocclusion, (6) loss of more than one tooth, (7) history of maxillofacial trauma, (8) pregnancy, (9) presence of contraindication for MRI, (10) presence of rheumatism, rheumatoid, and other systemic disease history, and (11) the use of drugs with action on the central nervous system, such as antidepressants.

### 2.2. Data Collection

The data collection process consists of the following four phases:

First, we used the State-Trait Anxiety Inventory (STAI) and the Beck Depression Inventory-Ⅱ (BDI-Ⅱ) to assess the anxiety and depression levels of all subjects.

Second, the TMJ status of all subjects was assessed under the guidance of RDC/TMD. The assessment included history of TMD, the palpation of the TMJ, and muscle, joint clicks, and the maximum mouth opening, both in the static position and the forward position. Based on the results of examination, the subjects who did not meet the requirements were excluded.

Third, according to the widely accepted SB diagnostic criteria proposed by the American Academy of Sleep Medicine (AASM) [15], potential SB subjects were screened and referred to Tianjin Medical University General Hospital for a lab-based PSG, which confirmed the eligibility of the subject to be a participant in the study group. These sleep bruxers were divided into three groups based on Molina’s criteria [16], among which nine presented mild (M), eight demonstrated moderate, and two showed severe bruxism. To facilitate statistical analysis, the last two subgroups were combined into the moderate to severe group (MS). In addition, the history (years) of SB was recorded.

Finally, the MR and CBCT images of bilateral TMJ were acquired.

MR imaging was collected by 3.0 Tesla Magnetom equipment (Siemens, Germany) with a 64-channel headband at the Tianjin Medical University General Hospital. The scanning process was as follows: Firstly, we obtained the positioning images. Then, in the closed mouth position, the alignment line was perpendicular to the long axis of the condyle, and the proton density-weighted turbo spin echo sequence (PDW-TSE) and the T2-weighted turbo spin echo sequence (T2W-TSE) in the oblique sagittal plane were scanned, and twenty-two images of the bilateral TMJ were obtained, respectively. Moreover, the alignment line was parallel to the long axis of the condyle, and the PDW-TSE sequence was scanned to obtain 13 images of the TMJ in the oblique coronal. Finally, the PDW-TSE sequences were scanned in the opened mouth position in the oblique sagittal plane and the oblique coronal plane, and the non-magnetic oral device was used to stabilize the opening position to reduce motion artifacts. All the scanning parameters of MRI are shown in Table 1. All the slice thicknesses (ST) = 2 mm.

CBCT imaging was performed at the Stomatology Hospital of Tianjin Medical University by using the KaVo 3D exam equipment of Germany’s KaVo Shengbon company, requiring all the subjects to be scanned at the intercuspal position. The scanning voltage was 120 KV, the exposure time was 7.0 s, the tube current was 5 mA, the reconstructed layer thickness was 0.1 mm, the image reconstruction time was 27 s, and the voxel was 0.25 mm^3^. The FOV was a value of uncertain size, which would be adjusted according to the facial size of the subjects, including complete bilateral TMJ, and should be as small as possible. The Invivo 5.0 software (Anatomage, San Jose, California, USA) was used for three-dimensional reconstruction of those DICOM data, and then for the acquired data’s measurement and data analysis.

### 2.3. Assessment of the MR and CBCT Images

**Figure 1:**
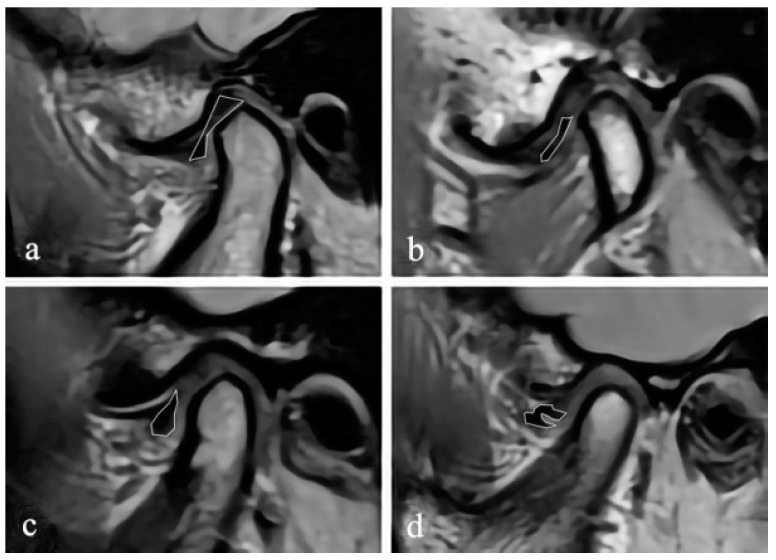
Disc configuration. Oblique sagittal proton density MRI showing the classification of disc configuration: (**a**) biconcave, (**b**) biplanar, (**c**) convex, and (**d**) folded.

**Figure 2:**
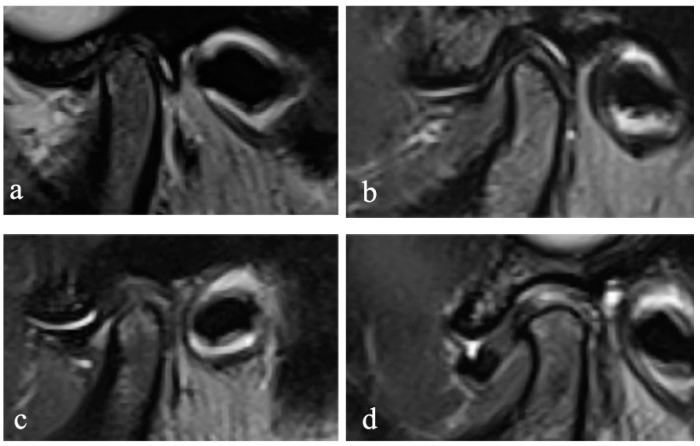
Joint effusion. Sagittal T2-weighted image MRI showing: (**a**) no fluid signal, (**b**) dots or lines of bright signal, (**c**) bands of bright signal, and (**d**) pooling of bright signal on the TMJ.

The MR images were randomly numbered and evaluated by using Radiant 5.0.0 software (Poznan, Poland) on the same laptop, and the information of the subjects, including the disc position, disc configuration, and JE, were hidden. In the closed and open mouth positions, the maximum section of the articular disc shown on the oblique sagittal PDW image was selected to evaluate the configuration of the disc. According to the method proposed by Raweewan [17], the disc configuration was divided into four types: biconcave, biplanar, convex, and folded (Figure 1). The biplanar, convex, and folded types were defined as deformation. Combined with the oblique sagittal and the oblique coronal PDW images, the position of the disc was described by Tasaki’s method [18]. Furthermore, JE was classified into 0–3 levels (Figure 2) on the oblique sagittal T2W images in the closed mouth position, according to the criteria proposed by Segami et al. [19]. The degree of JE was dichotomized into two groups for intra-group statistical analysis: JE-, grade 0 or 1, and JE+, grade 2 or 3.

**Figure 3:**
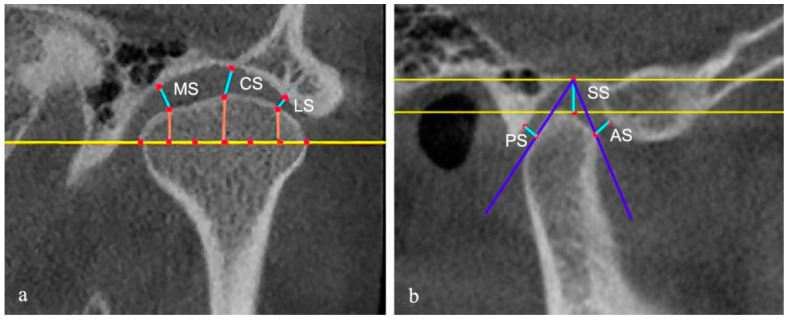
(**a**) The measurements of the lateral space (LS), central space (CS), and medial space (MS), and (**b**) the measurements of the anterior space (AS), superior space (SS), and posterior space (PS).

The authors evaluated the condyle and the joint spaces on the CBCT images. The sagittal and coronal images were reconstructed by an axial view showing the maximum mediolateral dimension of the condyle. The slice thickness was 1 mm. On the reconstructed CBCT images, as described by Ahmad et al., the surface of each condyle was classified as normal, flattening, erosion, osteophyte, subcortical sclerosis, and subcortical cyst [20]. Each possible change could appear alone or in combination in at least two sequential parasagittal sections. Considering that the increase of the diagnostic types may result in reduced reliability, we combined the types that were less common into one category for statistical purposes. The superior space (SS), posterior space (PS), and anterior space (AS) of joints were measured on the middle layer of the reconstructed sagittal images. The parameter of PS/AS was calculated to assess the anteroposterior relationship of the condylar to the fossa. Additionally, coronal images were also used to determine the position of the condyle. On the middle layer of the reconstructed coronal images, the lateral space (LS), central space (CS), and medial space (MS) were measured using the method of Ikeda [21] (Figure 3).

The two researchers (a master’s degree student and an associate professor) performed an initial assessment of MR and CBCT images of all subjects according to the above standards, respectively. After that, the researchers discussed the results of their respective assessments, and when differences existed, they read the images again and reached a consensus on the assessment criteria. Measurement of quantitative data such as joint space was performed three times at intervals of two weeks. The mean value of the three measurements was taken for statistical analysis.

### 2.4. Statistical Analysis

All statistical analyses were performed by using IBM SPSS Statistics 19.0 software (Chicago, USA). Each TMJ was identified as a separate unit for the purpose of statistical analysis. All the parameter data were tested for normality. Parameter statistics were performed only after they were in line with normal distribution. The McNemar test and the paired *t*-test were performed on the left and right TMJs of the same patients within the intra-group comparison. Meanwhile, the two-sample *t*-test, Pearson chi-square or Fisher’s exact test, and the Mann–Whitney U test were used to compare TMJ imaging characteristics of definite SB patients and asymptomatic non-bruxers. The variables used for comparison included the disc configuration, disc position, JE, joint space, and condylar bony changes. The inter-observer reliability was evaluated by the Kappa test, and the intra-observer reliability was assessed by the intraclass correlation coefficient (ICC). All statistical tests were two-tailed with a significance level of alpha = 5% (*p* < 0.05).

## 3. Results

In this study, the intra-observer reliability was considered completely reliable (ICC > 0.9), and the inter-observer reliability consistency was moderate (JE K = 0.700, disc configuration at the closed position K = 0.645, disc position K = 0.618, condylar bony changes K = 0.607).

The characteristics of all subjects are shown in Table 2. There was no statistically significant difference in age, gender, education level, BMI, anxiety, and depression levels between the two groups (*p* > 0.05).

In the results from the total 78 joints, a statistically significant difference was found between the 2 groups in disc configuration for the closed mouth position (*p* < 0.05, Table 3), whereas no significant difference was found in disc configuration for the opened mouth position (*p* > 0.05, Table 3).

**Figure 4:**
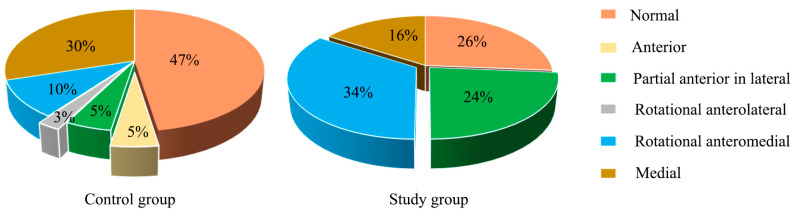
The proportion of disc displacement in the closed mouth position between the study group and the control group. The statistical results: x^2^ = 16.338, *p* = 0.002.

When combined with the sagittal and coronal MRI to assess the disc position in the closed mouth position, the disc displacement in the study group was significant compared to the control group (*p* < 0.05, Figure 4). The proportion of rotational anteromedial disc displacement (34%) and partial anterior in the lateral part of the joint (24%) increased in the study group. Overall, the prevalence of disc displacement in the study group was up to 74%, compared with 53% in the control group, of whom the most common could be observed as medial disc displacement (30%).

**Figure 5:**
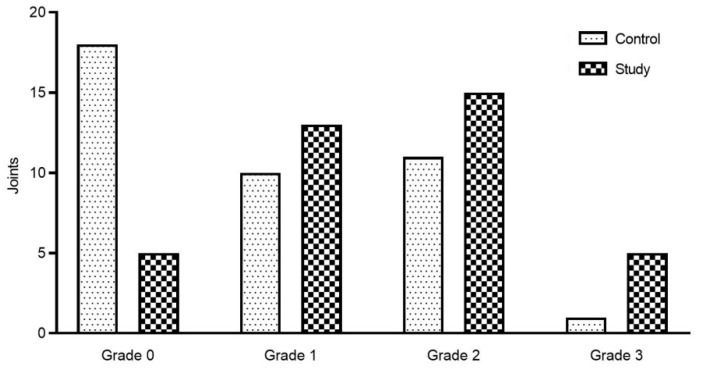
Joint effusion between the study group and the control group. The statistical results: Z = −2.974, *p* = 0.003.

JE was found in joints of the study group as well as in the control group on T2W images. The grade of JE in the study group was higher than that in the control group (*p* < 0.05, Figure 5). A statistically significant association was found between the history (years) of SB and the grade of JE (*p* = 0.005, Table 4). Furthermore, we also found that the prevalence of disc deformation, disc displacement, and JE+ was not significantly different between the left and right sides in the study group using the McNemar test (*p* > 0.05, Table 4).

**Figure 6:**
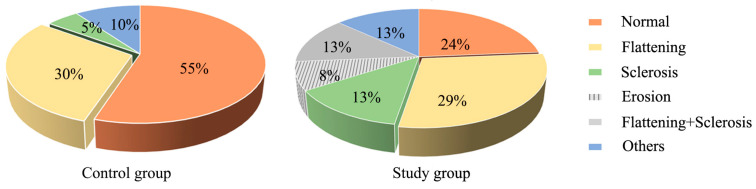
The proportion of condylar bony changes between the control group and the study group. The statistical results: x^2^ = 14.313, *p* = 0.007.

CBCT images of 78 TMJs were evaluated in 39 subjects (19 of whom were sleep bruxers and the other 20 were asymptomatic). As shown in Figure 6, a significant difference in the prevalence of condylar bony changes was also found between the study and control groups (*p* < 0.05). The results of the McNemar test showed that the prevalence of condylar bony changes was not significantly different between the left and right sides in the study group (*p* = 0.375, Table 5).

**Figure 7:**
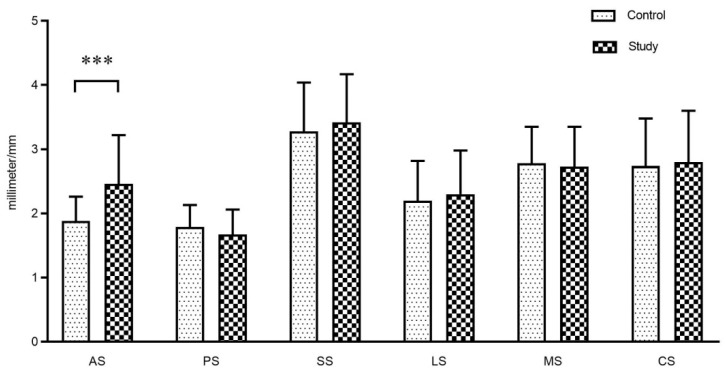
The size of the joint space between the study group and the control group. AS: anterior space, PS: posterior space, SS: superior space, LS: lateral space, MS: medial space, CS: central space. Results are shown as mean ± SD. *** *p* = 0.000.

**Figure 8:**
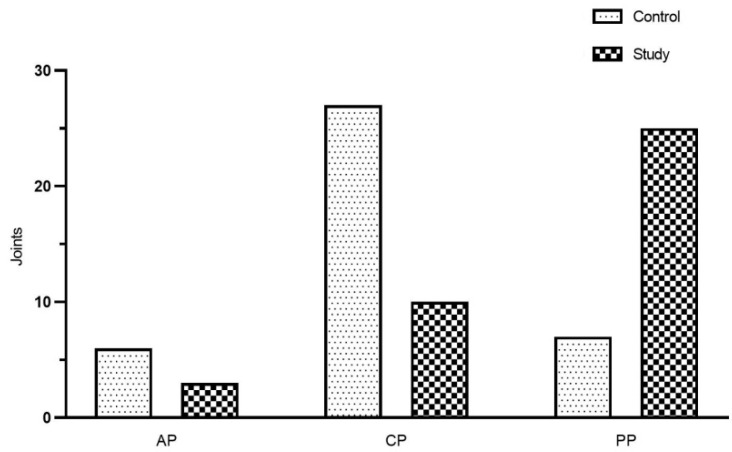
The position of the condyle between the study group and the control group (sagittal view). AP: anterior position, CP: central position, PP: posterior position. The statistical results: x^2^ = 19.198, *p* = 0.000.

The study also found that AS was significantly larger in the study group than that in the control group (*p* < 0.05), but there were no statistical differences between the two groups in other measurements (*p* > 0.05). Figure 7 shows the mean value of joint space between the two groups. The detailed distribution of the condyle position in the fossa (sagittal view) is shown in Figure 8 according to the results of ln (PS/AS). The results of the paired *t*-test showed that MS of the left TMJ decreased, and LS increased, compared with the right side in the study group (*p* < 0.05, Table 6), and no statistical difference was found in the control group (*p* > 0.05).

The chi-square analysis of the SB subgroup revealed no significant difference between the M group and the MS group in terms of JE (*p* = 0.330), disc configuration (*p* = 0.188), disc location (*p* = 0.144), condyle position (*p* = 0.783), or the condylar bony changes (*p* = 0.260), which is shown in Table 7.

## 4. Discussion

A previous study suggested that the prevalence of SB was the highest among young people, and declined with age [5]. Therefore, in this study, the age of the subjects was between 21 and 32 years old, and most of them were college students. For the depression and anxiety levels, the results of the study showed that there was no statistical difference between the study and the control groups. Similarly, Maluly et al. [22] also reported no difference in anxiety levels between the non-bruxers and the sleep bruxers. In contrast, Gungormus and Erciyas [23] found that the bruxers were more likely to be anxious and depressed, although they did not distinguish the type of bruxism. In the etiology of TMD, psychological factors cannot be neglected, and the anxiety and depression levels of the study group did not change significantly, which can exclude the interference of psychological factors on the results.

MRI has the advantages of being non-invasive, radiation-free, and having high-precision, and can accurately determine the disc position, disc configuration, and JE [24], while CBCT has the advantages of lower cost, lower radiation, and provides high-resolution multiplanar images of the TMJ [25]. Therefore, this study adopted 3.0T MRI and CBCT to evaluate the characteristics of TMJ soft and bone tissues in sleep bruxers, respectively. The consistency between observers in this study was not excellent, which is inseparable from the image blur caused by movement. In addition, the intra-observer agreement was higher than the inter-observer agreement in the present study, as expected. Many factors, such as background experiences and the ability to identify landmarks according to the definitions, also can affect the observers’ performance.

As is known, the disc is a biconcave fibrous cartilage structure, located between the mandibular condyle and the temporal bone, which can absorb the loads generated during TMJ movement. SB is a common source of micro-trauma in TMJ, often resulting in the deformation of the disc, and it has been shown to be a risk factor for the development of biplanar and hemi-convex disc morphology [24,26]. In this study, the proportion of disc deformation was higher in the study group in the closed mouth position, among which, the proportion of the convex and biplanar discs increased. Whereas during the process from the closed to the opened mouth position, most of the deformed discs in both groups returned to the original biconcave disc, with only a small portion remaining abnormal. These results indicated that the configuration of the discs was significantly related to the position of the mandible and was also closely associated with the biomechanics of the joint. This was consistent with the findings of Giozet et al. [26], which showed that the mouth position could influence disc morphology.

For the closed mouth position, the posterior band of the disc is normally located directly above the condyle, approaching twelve o‘clock. When its position changes, it is defined as disc displacement. A study has shown that the grinding activities were significantly associated with disc displacement [27]. This is consistent with the results of the current study. On the one hand, SB was considered an asymmetrical lateral movement of the mandible, in which the discs on both sides moved with the condyles. That pattern of movement might damage the lateral attachments of the discs. Similarly, it was also observed that the maximum shear stress of sleep bruxers was located on the lateral part of the disc, which might destroy the connection (lateral attachment) between the disc and the condyle, in turn causing the dislocation of the disc [6]. On the other hand, SB was a common source of micro-trauma in the TMJ, and repeated trauma could lead to repeated TMJ soft tissue damage, persistent inflammation affecting the collagenous structure of the disc, leading to deterioration in the mechanical properties of the disc, which might be an important factor in disc displacement [28]. Furthermore, the SB activity changed the lubrication of the joint and caused a change in friction, leading to a degenerative change in the disc that caused the disc to gradually displace [29]. The present study observed that the study group had a higher prevalence of disc displacement than the control group, in which the proportion of rotational anteromedial disc displacement and the partial anterior disc displacement in the lateral part of the joint increased. It may be related to the destruction of the lateral attachment during SB and the changes in the structure and performance of the disc. Hitherto, the clinical significance of disc displacement in the coronal plane is still ambiguous, and it is speculated that medial disc displacement may be related to the lateral movement of the condyle and the weak lateral attachment. In most previous studies, selecting only a central image to evaluate the disc position might lead to a false negative result, leaving out part of the disc displacement. In contrast, the current study reflected the disc position more accurately and comprehensively by assessing its position on all images of the oblique sagittal and coronal view.

JE seen on T2-weighted images was severe in the study group. The study also found that the time (years) of SB history was correlated with the grade of JE, which means the grade of JE would become higher with the increase of SB history. This result has rarely been reported before. JE was thought to be an inflammatory process [12]. A previous study suggested that the concentration of proteins and inflammatory cytokines increases in joints with effusion [30]. Attentively, the large amount of muscle activity accompanying SB caused abnormal mechanical stress in the joint, leading to the accumulation of irritants such as free radicals and nitric oxide in the tissue fluid, thereby resulting in JE [31]. Furthermore, when the mandible was in the resting position and the pressure was lower, the articular surfaces absorbed small amounts of synovial fluid, and once when the pressure was increased during clenching, the articular surfaces would release the synovial fluid; therefore, prolonged pressure would also cause JE [32]. Another study considered that JE might be the accumulation of synovial fluid and has no direct relationship with inflammation, and also proposed that the disc displacement may interfere with the physiological circulation of synovial fluid, thus leading to JE [33]. Currently, it is thought that the JE is related to the disc displacement, but not to the disc configuration [34]. This view could explain why JE was also observed in the control group of this study. This is consistent with the previous findings that JE is most common in symptomatic patients, but can also be found in some asymptomatic subjects [35].

Currently, moderate loading is known to promote anabolism of the TMJ; conversely, excessive and repetitive loading will lead to adaptation or degeneration of the TMJ, including hard and soft tissues. CBCT images in this study confirmed a higher prevalence of condylar bony changes in the study group when compared to the control group, which was consistent with the previous study [12]. Another study has suggested that excessive loading would change the original shape of the condyle, resulting in rounded contouring of the surface flattening, cortical thickening, and subchondral sclerosis [36]. Flattening and subcortical sclerosis were considered to be the signs of TMJ remodeling [20], and these changes in the bone may be the result of chronic overloading of the TMJ, such as SB, which may result in bone absorption and flattening of the rounded articular surface due to the repetitive mechanical overloading it produces [37]. Likewise, Israel et al. [38] reported that the TMJ loading caused by SB was the cause of TMJ osteoarthritis under arthroscopy. Excessive loading could trigger a series of events, such as the production or release of free radicals, cytokines, fatty acid catabolites neuropeptides, and matrix-degrading enzymes, that degraded the matrix. When the loading is beyond the individual’s ability to adapt, degeneration might occur in the articular surface [39]. In this study, the proportion of flattening combined with subcortical sclerosis in the study group increased, while such change was not observed in the control group, suggesting that condyle bone could undergo corresponding changes after absorbing the abnormal loads. In addition, the condyle bone might also change in the control group, with the flattening being more common, indicating that the condylar surface could be flattened under the influence of functional or parafunctional activities, suggesting that simple flattening may be a normal adaptive response to the loading.

In radiology, the condyle position can be determined by the size of the joint space between the condyle and the fossa. It is well-known that the superior space (SS) is the largest in the joint space. Similarly, the SS was also the largest in the current study. Besides, the anterior space (AS) in the study group was significantly larger than that in the control group, and the condyle was usually located in the posterior position of the fossa, which is consistent with the findings of Cho and Jung [40]. Up to now, the clinical significance of the condyle position in the TMJ has been controversial, and the current prevailing view is that the condyle position can be used as a valuable diagnostic aid for TMD [41]. One study advocated that the condyle position was related to TMD, and the posterior position was more common in patients with severe TMD [42], while another study had not demonstrated a correlation between the condyle position and TMD [43]. Three-dimensional finite element analysis showed that the condylar displacements after 10 min of clenching were 0.21 mm and 0.44 mm in the asymptomatic and symptomatic joints, respectively [44]. Similarly, Hirose et al. [45] showed that clenching over a long period caused the condyle to move posteriorly. The current results implied that the condyles of SB patients were more prone to be posterior and more prominent than those who were asymptomatic, suggesting that in the clinical treatment of SB-TMD patients, the splint could be appropriately used to guide the condyle forward.

It is expected that there was no difference in the control group when comparing the left and right TMJs, whether it was soft tissue or bone tissue, indicating that the two sides of the TMJs were basically symmetrical, while the medial space (MS) of the left TMJ decreased and the lateral space (LS) increased compared with the right TMJ in the study group, indicating that the position of the two sides of the condyles was not symmetrical in the coronal section. Del Palomar [6] has also pointed out that the working side and non-working side condyles move in different directions when SB attacks, i.e., the working side condyles move slightly in the posterior and lateral directions, meanwhile, the non-working side condyles move in the anterior and medial directions. It is a reasonable explanation for the difference in the position of the condyles in the coronary position in sleep bruxers. Furthermore, these movements are the results of masticatory muscle contractions, which indicates that the position of the condyle in the articular fossa is closely related to the masticatory muscles, whereby if the left and right masticatory muscles contract inconsistently, the mandible may be in an asymmetric position. After subgroup analysis for these sleep bruxers, the study found that the severity of SB was not related to the imaging performance of the TMJ, implying that the presence of sleep bruxism could affect the TMJ, whether severe or not. The lack of statistical significance was probably due to the small sample size. It should be clearly pointed out that the severity of SB was classified according to the subjective symptoms reported by sleep bruxers.

There are some limitations in the current study. First, due to the limitations of time and cost, the asymptomatic group did not exclude SB through PSG. Due to the limited resources of PSG, MRI, and CBCT, the small sample size was the second limitation of the study. Third, MRI and CBCT were performed by different radiologists, but all were performed according to the same standard. Finally, the position of the mandible during scanning was the most difficult to control and may affect the results of the investigation. To improve the accuracy, most of the subjects we recruited were engaged in the stomatology field.

## 5. Conclusions

It was observed that a higher prevalence of disc deformity, disc displacement, JE, and condylar bony changes occurred in joints of sleep bruxers in the closed mouth position. On MR images of TMJs in sleep bruxers, the abnormality could be characterized by the convex and biplanar disc, rotational anteromedial disc displacement, and a higher grade of effusion. On CBCT images, except for the changes in the condylar bone, the positions of the left and right condyles in sleep bruxers were asymmetrical, and the condyle was significantly in the posterior position. In the clinical treatment of SB-TMD patients, the splint could be appropriately used to guide the condyle forward. Appropriate treatment such as physiotherapy may be used to reduce the formation of or promote the absorption of fluid accumulation. However, this study did not find a relationship between the severity of SB and the reconstruction of the TMJ, so it is important to increase the sample size for further study.

## Figures and Tables

**Table 1:** All scanning parameters of MRI.

Scanning Parameters	The PDW-TSE in the Oblique Sagittal	The PDW-TSE in the Oblique Coronal	The T2W-TSE
TR (ms)	3000	3200	4050
TE (ms)	32	32	75
FOV (mm)	140	160	160
Voxel size (mm)	0.4 × 0.4 × 2.0	0.5 × 0.5 × 2.0	0.5 × 0.5 × 2.0

TR: reverse time, TE: echo time, FOV: field of view.

**Table 2:** The distribution and percentage of characteristics, anxiety and depression levels, and oral habits in the study and the control group.

Variables	Control	Study	*p*
Gender			
Male, n (%)	4 (20%)	4 (21.1%)	1.000
Female, n (%)	16 (80%)	15 (78.9%)	
History of orthodontic treatment			
Yes, n (%)	2 (10%)	3 (10.5%)	0.661
No, n (%)	18 (90%)	16 (89.5%)	
Chewing hard food			
Yes, n (%)	3 (15%)	4 (21.1%)	0.695
No, n (%)	17 (85%)	15 (78.9%)	
Age (years)	25.9 ± 2.6	25.2 ± 2.2	0.374
BMI (kg/m^2^)	20.5 ± 3.1	20.9 ± 3.1	0.644
SAI (scores)	32.1 ± 7.6	34.5 ± 10.8	0.421
TAI (scores)	36.2 ± 5.0	36.9 ± 7.9	0.707
BDI (scores)	5.1 ± 4.0	4.7 ± 4.5	0.819
Education level (years)	18.3 ± 0.85	17.6 ± 2.5	0.332

BMI: body mass index (kg/m^2^), SAI: State Anxiety Inventory, TAI: Trait Anxiety Inventory, BDI: Beck Depression Inventory.

**Table 3:** The distribution of disc configuration between the study group and the control group.

Position	Group	The Configuration of the Articular Disc				*p*
		Biconcave (%)	Biplanar (%)	Convex (%)	Folded (%)	
Closed	Control	72.5	12.5	10	5	0.043 *
	Study	42.1	21.0	29	7.9	
Opened	Control	85	15	-	-	0.874
	Study	81.6	15.8	-	2.6	

* *p* < 0.05.

**Table 4:** The association between the grade of joint effusion and the history of sleep bruxism.

The Grade of JE	TMJ (n)	History of Sleep Bruxism (years)	*p*
Grade 0	5	3.20 ± 1.64	0.005 **
Grade 1	13	5.62 ± 3.23	
Grade 2	15	6.33 ± 2.64	
Grade 3	5	8.40 ± 1.82	

** *p* < 0.01. JE: joint effusion.

**Table 5:** A comparison of qualitative data on left and right TMJs in sleep bruxers.

	Left (%)	Right (%)	*p*
Disc configuration (closed)			
biconcave	52.6	31.6	0.219
deformation	47.4	68.4	
Disc displacement			
normal	26.3	26.3	1.000
displacement	73.7	73.7	
Effusion			
+	52.6	52.6	1.000
−	47.4	47.4	
Condylar bony change			
normal	31.6	15.8	0.375
abnormal	68.4	84.2	

**Table 6:** The comparison of joint space between the left and right TMJs in sleep bruxers.

Variables	Left (Mean ± SD)	Right (Mean ± SD)	*p*
AS (mm)	2.46 ± 0.77	2.45 ± 0.77	0.955
PS (mm)	1.67 ± 0.42	1.67 ± 0.38	0.970
SS (mm)	3.45 ± 0.83	3.39 ± 0.67	0.619
LS (mm)	2.58 ± 0.69	2.01 ± 0.56	0.001 **
MS (mm)	2.48 ± 0.61	2.98 ± 0.53	0.006 **
CS (mm)	2.90 ± 0.87	2.69 ± 0.73	0.175

** *p* < 0.01. AS: anterior space, PS: posterior space, SS: superior space, LS: lateral space, MS: medial space, CS: central space.

**Table 7:** A comparison of the TMJ between the M group and the MS group.

	M Group (n = 9)		MS Group (n = 10)	
	Left	Right	Left	Right
Disc configuration (closed)				
biconcave	6	4	4	2
deformation	3	5	6	8
Disc displacement				
normal	3	4	2	1
displacement	6	5	8	9
Effusion				
+	5	6	5	4
−	4	3	5	6
Condylar bony change				
normal	4	2	2	1
abnormal	5	7	8	9
Condyle position (sagittal)				
anterior	1	0	1	1
central	2	2	2	4
posterior	6	7	7	5

M: mild sleep bruxism, MS: moderate to severe sleep bruxism.

## Data Availability

The data presented in this study are available upon request from the corresponding authors. The data are not publicly available due to ethical reasons.
